# Multiomics analyses of Jining Grey goat and Boer goat reveal genomic regions associated with fatty acid and amino acid metabolism and muscle development

**DOI:** 10.5713/ab.23.0316

**Published:** 2023-11-02

**Authors:** Zhaohua Liu, Xiuwen Tan, Qing Jin, Wangtao Zhan, Gang Liu, Xukui Cui, Jianying Wang, Xianfeng Meng, Rongsheng Zhu, Ke Wang

**Affiliations:** 1 Institute of Animal Science and Veterinary Medicine, Shandong Key Lab of Animal Disease Control and Breeding, Shandong Academy of Agricultural Sciences, Jinan 250100, China; 2 Key Laboratory of Livestock and Poultry Multi-omics of MARA, Jinan 250100, China; 3 Shandong Animal Husbandry General Station, Jinan 250100, China

**Keywords:** Goat, Meat Quality, Muscle Development, RNA-seq, Selective Signature

## Abstract

**Objective:**

Jining Grey goat is a local Chinese goat breed that is well known for its high fertility and excellent meat quality but shows low meat production performance. Numerous studies have focused on revealing the genetic mechanism of its high fertility, but its highlighting meat quality and muscle growth mechanism still need to be studied.

**Methods:**

In this research, an integrative analysis of the genomics and transcriptomics of Jining Grey goats compared with Boer goats was performed to identify candidate genes and pathways related to the mechanisms of meat quality and muscle development.

**Results:**

Our results overlap among five genes (*ABHD2*, *FN1*, *PGM2L1*, *PRKAG3*, *RAVER2*) and detected a set of candidate genes associated with fatty acid metabolism (*PRKAG3*, *HADHB*, *FASN*, *ACADM*), amino acid metabolism (*KMT2C*, *PLOD3*, *NSD2*, *SETDB1*, *STT3B*, *MAN1A2*, *BCKDHB*, *NAT8L*, *P4HA3*) and muscle development (*MSTN*, *PPARGC1A*, *ANKRD2*). Several pathways have also been detected, such as the FoxO signaling pathway and Apelin signaling pathway that play roles in lipid metabolism, lysine degradation, N-glycan biosynthesis, valine, leucine and isoleucine degradation that involving with amino acid metabolism.

**Conclusion:**

The comparative genomic and transcriptomic analysis of Jining Grey goat and Boer goat revealed the mechanisms underlying the meat quality and meat productive performance of goats. These results provide valuable information for future breeding of goats.

## INTRODUCTION

The goat (*Capra hircus*), one of the most important livestock species, has been domesticated about 10,500 to 9,900 years ago [1]. It provides a range of products including milk, meat and fiber and plays economically important roles in human productive activity [2]. China has a long history of goat domestication and rich resources on goat breeds [3]. Jining Grey goat (JG) is one Chinese local goat breed, famous for high fertility and excellent meat quality, but it has small body size and low meat production performance. Numerous reports have focused on revealing the genetic mechanism of its high fertility [4], but its highlighting meat quality and muscle growth mechanism still need to be studied.

Meat quality is a complex trait affected by genetics, age, nutrition, and gender [5]. Fat deposition on carcasses has a significant impact on meat yield and consumer perception of eating quality [6]. Lipids and fatty acids are some of the main constituents and flavor contributors in meat [7]. Interestingly, breed-specific differences have been reported in the fat composition of meat [8]. Different expression levels of enzymes involved in lipid metabolism have been hypothesized to account for such distinctions [9]. The oxidation of fatty acids is another way fat affects meat flavor [10]. Research on flavor metabolites in goat meat showed that among the top two most abundant metabolites in meat from JG were pyranodelphinin A and lysoPC (17:0), which were more abundant than those in meat from Boer goat (BG). In addition, Amyl salicylate and hexyl salicylic acid levels in JG meat are significantly higher than in BG meat. Among fatty acids and their metabolic derivatives, 3-hydroxy-9Z-octadecenoylcarnitine and tiglylcarnitine are significantly more enriched in JG- than BG-derived goat meat [7]. The variety and contents of amino acids are also closely related to the flavor of meat [11]. Our previous phenotypic study revealed that the muscle fiber diameter of JG is narrower than that of BG (p<0.05). In addition, the contents of essential amino acids, delicious amino acids, and sweet amino acids in the *longissimus thoracis* of JG are higher than those in the *longissimus thoracis* of BG [12].

In livestock and poultry, skeletal muscles are the main sources of animal protein, and the growth and development of skeletal muscle directly influence animal meat quantity and quality [13]. However, the previous phenotypic study showed that the slaughter performance of JG was significantly lower than that of BG, and there were significant differences in live weight, carcass weight and net meat weight (p<0.01) [12]. Thus, it is equally significant to investigate the genetic mechanism of muscle development for the breeding of JG.

The goat genome is the research basis for the protection and utilization of goat resources, and the whole genome sequencing (WGS) is important for the continued breeding and improvement of goat breeds [14]. Besides, RNA sequencing (RNA-Seq) is a popular and efficient technology that offers a plethora of genetic data such as the differentially expressed genes (DEGs), and used to investigate the mechanisms of meat quality in multiple species, such as sheep [11] and cattle [15]. Integrative analysis of WGS and transcriptome is one of the effective methods for identifying functional genes in biological processes. The differential expression on the transcriptional level is a validation to genomic selection signals. In addition, WGS could also revealed key variations (including single nucleotide polymorphisms [SNPs] and insertions and deletions [INDELs]) in the DEGs. This method had been used in multiple reported studies of animals [16]. Therefore, in the present study, integrative analysis of the genomics and transcriptomics of JG compared with BG was performed with the aim of investigating candidate genes and signaling pathways related to fatty acid and amino acid metabolism and muscle development. This work will help to reveal the mechanisms underlying goat meat quality and muscle development traits.

## MATERIALS AND METHODS

### Animal care

The experimental protocols were reviewed and approved by Institutional Animal Care and Use Committee of Institute of Animal Science and Veterinary Medicine, Shandong Academy of Agricultural Sciences (IACC20060101).

### Animals and sample collection

Whole blood was sampled from jugular vein for 12 JG, half of which came from the Caoxian Zhengdao Animal Husbandry Technology Co., Ltd. (Heze, China), while the other half came from Jining Grey Goat Original Seed Farm.

Three genetically unrelated male JG individuals were selected from Caoxian Zhengdao Animal Husbandry Technology Co., Ltd., and three genetically unrelated male BG individuals were selected from a purebred herd. All individuals were raised in the experimental sheep farm of Shandong Academy of Agricultural Sciences, and raised from 10 months old to 12 months old under the same feeding conditions. The average initial weight of JG and BG was 17.02 kg and 33.9 kg, respectively. The terminal weight was 18.8 kg and 37.8 kg, respectively. To get samples of the *longissimus thoracis* for transcriptome analysis, the animals were promptly put to death. Following collection, the fresh tissues were promptly flash-frozen in liquid nitrogen and then preserved at −80°C until use.

### DNA extraction and genome sequencing

Whole blood samples were extracted with the E.Z.N.A. Tissue DNA kit for DNA (Omega Bio-Tek, Norcross, GA, USA). Genomic DNA (at least 3 μg) from each sample was used to construct a sequencing library with an average insert size of 450 bp. Sequencing was performed on the Illumina HiSeq X platform with 150 bp paired-end reads. Moreover, 10 publicly available genome sequences from BG were employed for comparative analysis [17].

### Alignment, variant discovery, and annotation

Following quality control by Trimmomatic (version 0.36) using the following parameters (LEADING:3, TRAILING:3, SLIDINGWINDOW:4:15, MINLEN:75) [18], each individual’s clean reads were mapped using BWA software to the goat reference genome (ARS1) [19]. With PICARD tools, duplicates of polymerase chain reactions were removed. The genome analysis toolkit (GATK) was used to realign INDELs [20]. Single nucleotide polymorphisms and INDELs were called with SAMTools [19]. For further analysis, only biallelic SNPs and INDELs with calling rates >90% and quality values >30 were retained. After filtering, ANNOVAR was used to annotate detected variations [21].

### Population genetics analysis

Pairwise genetic distances were calculated using the SNPs and the distance matrix was calculated by the software PHYLIP 3.68 [22]. The phylogenetic tree analysis was constructed using neighbour-joining method by MEGA11. Principal component analysis (PCA) was performed by EIGENSOFT [23].

### Selective sweep and candidate gene analysis

In this study, we conducted the WGS of 12 JG and collected publicly available whole genome data for 10 BG (6 from Australia and 4 from Korea). Regions of selective sweeps in the goat genome were searched using three approaches. First, the average pooled heterozygosity (*H*_P_) was calculated in 100-kb windows with half-step sliding, according to a previous report [24]. Then, the fixation index (*F*_ST_) values for individual SNPs between JG and BG and averaged the *F*_ST_ values across each 100-kb window were calculated [25]. After Z-transformation of the distribution of the *H*_P_ and *F*_ST_ values, cutoffs of Z(*H*_P_)_JG_ <–1.498 and Z(*F*_ST_) >1.773 were applied to the extreme ends of the distribution to obtain putatively selected windows. In addition, the cross-population extended haplotype homozygosity test (XP-EHH) for JG and BG were estimated using BG as a reference [26]. A threshold of XP-EHH >0.121 was used to identify selective sweep regions. Candidate genes within the identified regions (extending 50 kb up- and downstream) were identified by Ensembl gene annotation. In order to understand the biological functions of the identified candidate genes, KOBAS-i tools were used to perform the gene ontology (GO) enrichment and Kyoto encyclopedia of genes and genomes (KEGG) pathway analyses [27].

### cDNA library preparation and RNA-seq

Total RNA was extracted with Trizol reagent (Invitrogen, Shanghai, China) from muscle samples, according to the manufacturer’s protocols. The RNA quality and quantity were assessed using RNA Nano 6000 Assay Kit on Agilent 2100 Bioanalyzer (Agilent Technologies, Santa Clara, CA, USA). The mRNA was used as a template to synthesize cDNA. cDNA and second-strand cDNA were synthesized and purified to obtain the final library using a QiaQuick PCR kit (Qiagen, Valencia, CA, USA). After test by Agilent 2100 Bioanalyzer and Real-Time PCR System, the libraries were sequenced on an Illumina HiSeq 2000 (Beijing, China).

### Quality control, annotation, and differential expression analysis

After reads containing adapter, reads containing ploy-N and low-quality reads from raw data were removed, clean data were obtained for subsequent analysis. The clean reads were mapped to the goat reference genome. The gene expression levels were evaluated by quantifying reads per kilobase per million mapped reads (RPKM) values. False discovery rate (FDR) was used to assess the p-value in multiple tests. Fold changes (log2ratio) were estimated according to the normalized gene expression level in each sample. An FDR ≤0.001 and the absolute value of log_2_ratio ≥1 were used as the thresholds for identifying DEGs.

### Functional enrichment and protein-protein interactions network analysis of differentially expressed genes

The GO enrichment and KEGG pathway analyses of DEGs were performed using KOBAS-i tools [27]. Protein-protein interactions (PPI) network analysis was also performed on DEGs using STRING database and Cytoscape software.

## RESULTS

### Generation and analysis of multiomics datasets

**Figure 1:**
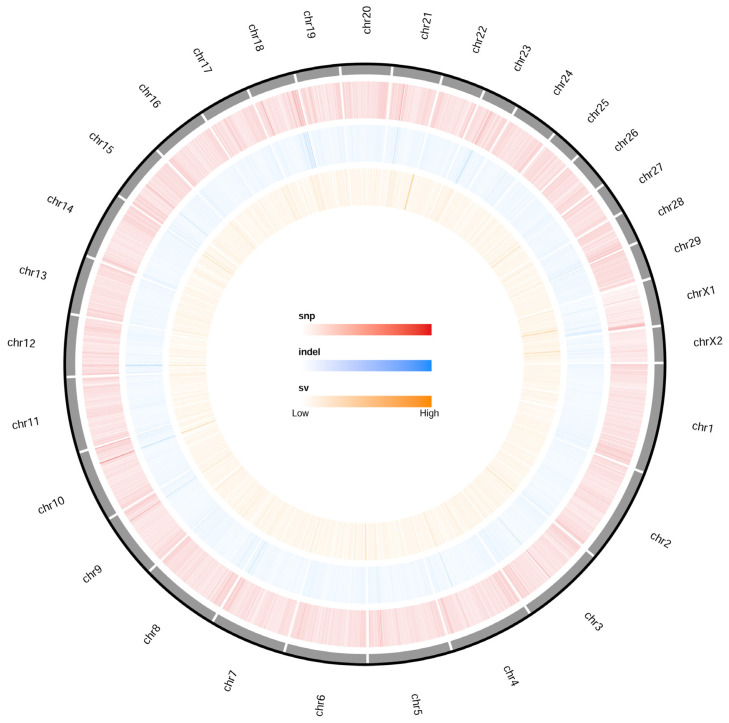
Circos plot of variations distribution in Jining Grey goat chromosome.

To characterize the distribution of sequence variation across the whole genome, we identified an average of 6.17 million filtered SNPs and 0.47 million insertion-deletion polymorphisms (InDels) per individual, with an average sequencing depth of 13.45% and 99.17% coverage of the goat reference genome (ARS1), for analysis (Figure 1; Supplementary Table S1, S2).

We also analyzed the gene expression profile of the *longissimus dorsi* muscle from the two goat breeds (Supplementary Table S3). The results revealed extensive gene expression in the muscle tissue of JG and BG (Supplementary Table S4). In the two libraries, there were 12,311 (55.52%) and 12,100 (54.57%) known reference genes expressed in the JG and BG samples, respectively, and 11,204 (50.53%) genes were identical between the two samples (Supplementary Table S5).

### Population structure analysis

Phylogenetic tree with SNP of each individual revealed that the two groups of BG, Korean Boer Goat (KB) and Australian Boer Goat (AB) gathered into a cluster, except for one individual AB4. A long genetic distance exists between most individuals of BG and JG (Supplementary Figure S1). Furthermore, a PCA of all 22 goat individuals also demonstrated that two groups of BG had close genetic relationships which are far from JG, except for AB4 (Supplementary Figure S2).

### Selection signal analysis and functional gene identification

**Figure 2:**
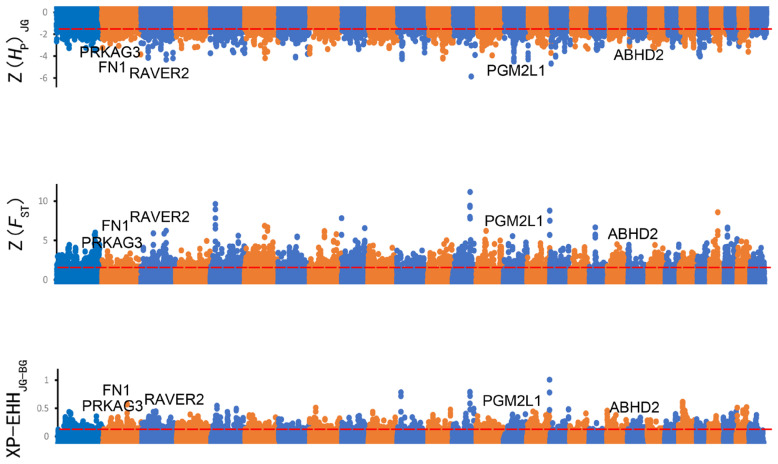
Manhattan plot of selective sweeps in Jining Grey goat. Z(*H*_P_), Z(*F*_ST_), and XP-EHH values were calculated in 100-kb windows with half-step sliding across all autosomes in Jining Grey goat.

**Figure 3:**
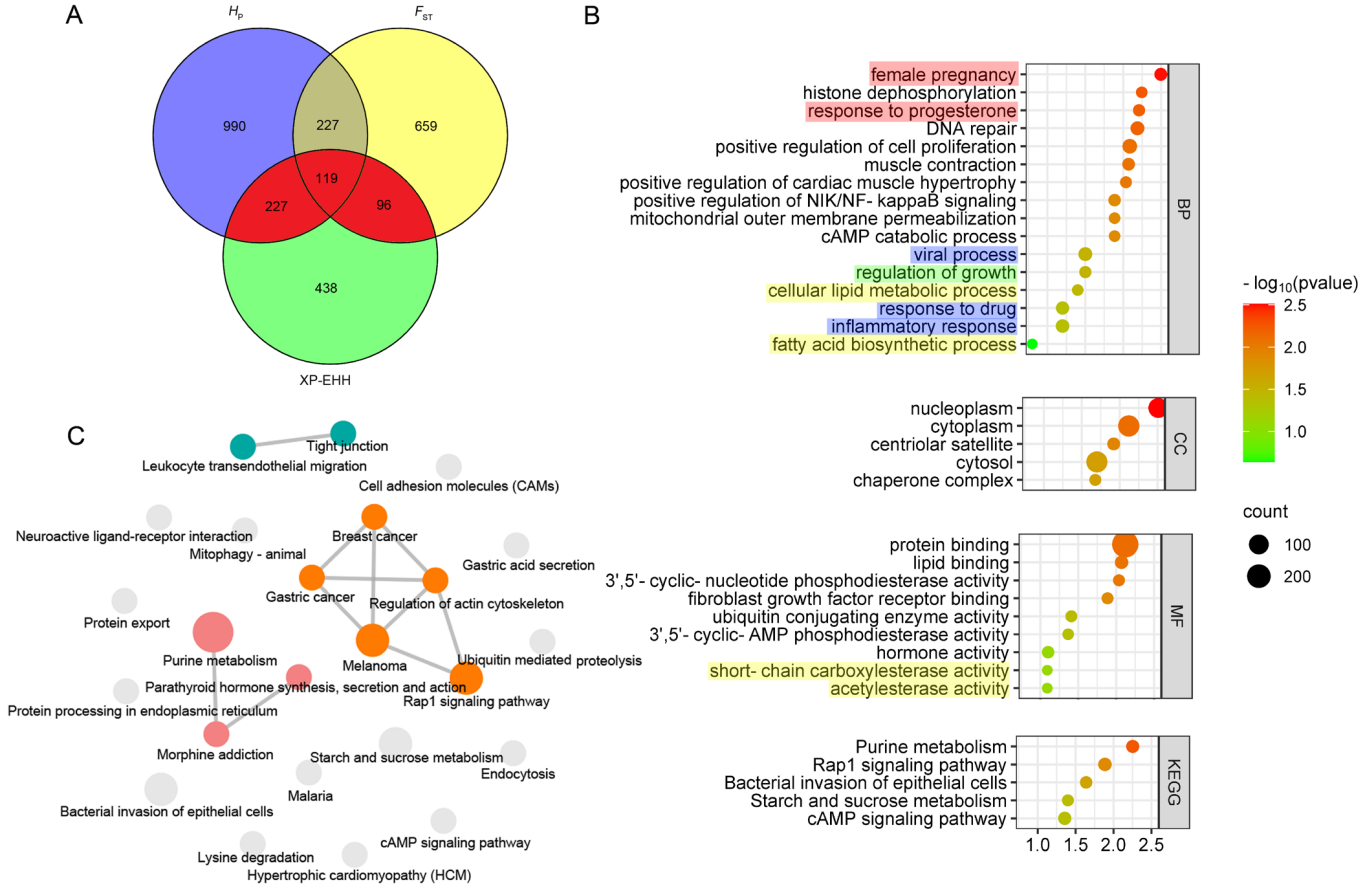
GO and KEGG enrichment analysis for the identified candidate genes. (a) A Venn plot showing numbers of overlapping candidate genes identified by at least 2 tests. In total, 442 candidate genes under positive selection of JG identified by at least two tests were shown in red. (b) GO enrichments involved in reproduction (in red shadow), immunity (in blue shadow), growth (in green shadow), and lipid metabolism (in yellow shadow) for the 442 candidate genes. (c) cirFunMap showing the enriched KEGG pathway terms. Interacting signal pathways are linked together. GO, gene ontology; KEGG, Kyoto encyclopedia of genes and genomes.

Selection signatures across the whole genome had been identified in JG and BG using *H*_P_, *F*_ST_, and XP-EHH (Figure 2). First, the *H*_P_ values of JG and the *F*_ST_ values between JG and BG were calculated, and 1,563 and 1,101 candidate genes were identified with cutoffs of the top 5% of Z(*H*_P_) and Z(*F*_ST_) (Z(*H*_P_)_JG_ <–1.498, Z(*F*_ST_) >1.773), respectively (Figure 2A, 2B; Supplementary Table S6). In addition, 880 genes (top 5% outliers, XP-EHH value >0.121) were found to be positively selected in the XP-EHH analysis (Figure 2C; Supplementary Table S6). Overall, 442 candidate genes under positive selection were identified in JG by at least two tests simultaneously (Figure 3A). Similarly, 594 overlapping genes were identified as candidate genes for BG, while 16 candidate genes were shared between JG and BG.

The GO enrichment and KEGG pathway analyses suggested the mechanism affecting meat quality traits and identify related candidate genes for JG (Figure 3). The GO analysis revealed that 442 candidate genes were significantly enriched in biological functions such as reproduction (female pregnancy, response to progesterone, maternal process involved in parturition), immunity (viral process, inflammatory response, response to drug), growth (regulation of growth, growth factor activity), and lipid metabolism (cellular lipid metabolic process, fatty acid biosynthetic process) (Figure 3B). We speculated that the outstanding meat quality of JG is related to lipid metabolism. Three candidate genes (*ABHD2*, *ABHD3*, *ZMPSTE24*) were associated with cellular lipid metabolic process and another two (*ABCD3*, *PRKAG3*) with atty acid biosynthetic process. Some significant KEGG pathways were also detected, and the enriched terms were visualized in cirFunMap (Figure 3C). Among these pathways, purine metabolism was the most significant, involving nine candidate genes (*NME4*, *ADCY3*, *ENPP3*, *RRM1*, *PRUNE1*, and PDE gene family members 4B, 7A, 7B, and 9A). In addition, melanoma, gastric cancer, breast cancer and other disease pathways were identified, and six candidate genes (*GADD45A*, *RBP3*, and FGF gene family members 5, 7, 8, and 21) were found to be related to all of the above pathways. Several other candidate genes were associated with amino acid metabolism pathways such as lysine degradation (*KMT2C*, *PLOD3*, *NSD2*, *SETDB1*), N-glycan biosynthesis (*STT3B*, *MAN1A2*), valine, leucine and isoleucine degradation (*BCKDHB*), alanine, aspartate and glutamate metabolism (*NAT8L*), and arginine and proline metabolism (*P4HA3*). Furthermore, the FoxO signaling pathway (*GADD45A*, *PLK3*, *PRKAG3*, *BNIP3*) and Apelin signaling pathway (*SPHK2*, *PRKAG3*, *ADCY3*) might play roles in lipid metabolism. The candidate gene *PRKAG3* participated in both pathways.

### Analysis of RNA-seq based differentially expressed genes and functional enrichment

**Figure 4:**
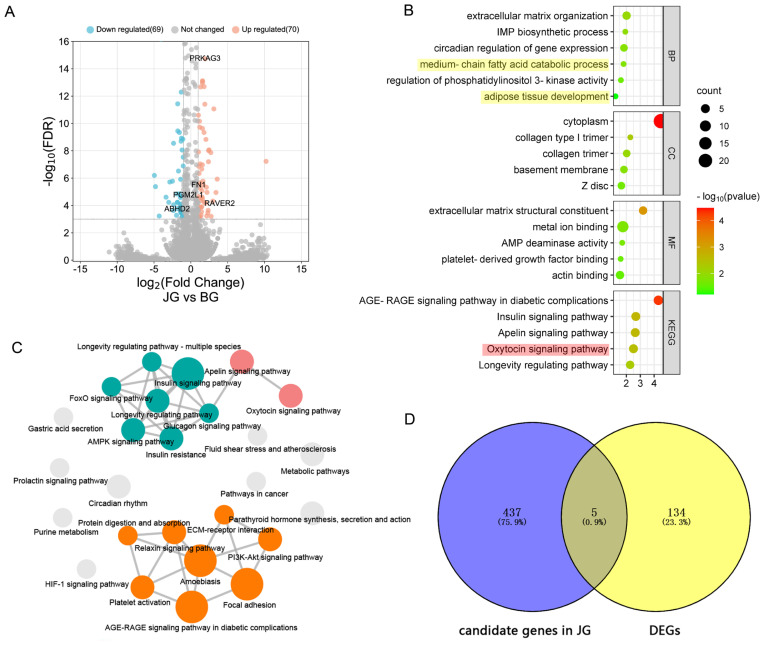
GO and KEGG enrichment analysis for DEGs. (a) Volcano plot of DEGs. Orange dots represent upregulated DEGs, green dots represent downregulated DEGs, and grey dots represent no significant change between groups. (b) GO enrichments involved in reproduction (in red shadow) and lipid metabolism (in yellow shadow) for DEGs. (c) cirFunMap showing the enriched KEGG pathway terms. Interacting signal pathways are linked together. (d) A Venn plot showing numbers of overlapping candidate genes identified by whole genome selection signal analysis and Analysis of RNA-seq based DEGs. GO, gene ontology; KEGG, Kyoto encyclopedia of genes and genomes; DEGs, differentially expressed genes.

Using a FDR ≤0.001 and an absolute value of the fold changes (log_2_ratio) ≥1 as the threshold values, 139 genes were found to be differentially expressed, including 70 upregulated and 69 downregulated genes in the JG library (Figure 4A). GO analysis revealed that DEGs were significantly enriched in biological functions such as muscle development (skeletal muscle cell differentiation, skeletal muscle atrophy, muscle tissue development, muscle organ development), adipose development (adipose tissue development, fat cell differentiation, brown fat cell differentiation), and lipid metabolism (medium-chain fatty acid catabolic process, fatty acid biosynthetic process, fatty acid beta-oxidation, fatty acid metabolic process, positive regulation of fatty acid oxidation) (Figure 4B). In the JG library, 21 DEGs were related to muscle development, among which the upregulation of *MTSN* might contribute to the lower slaughter performance of JG. Additionally, 18 DEGs related to lipid metabolism and the upregulation of *PRKAG3* and *FASN*, two important candidate genes related to meat quality, may contribute to the outstanding meat quality of JG. Four DEGs involved in the collagen biosynthetic process (*COL3A1*, *COL1A1*, *COL1A2*, and *COL15A1*) were upregulated in JG, which suggested that they are related to meat quality.

In addition, KEGG analysis was performed, and the enriched terms were visualized in cirFunMap (Figure 4C). Multiple pathways related to lipid metabolism were identified, such as the Apelin signaling pathway (*PLCB1*, *PRKAG3*, *EGR1*, *MYLK2*, *PPARGC1A*), FoxO signaling pathway (*FOXO1*, *INSR*, *PRKAG3*, *SGK1*), fatty acid metabolism (*HADHB*, *FASN*, *ACADM*), adipocytokine signaling pathway (*PRKAG3*, *PPARGC1A*), and PPAR signaling pathway (*FABP5*, *ACADM*). Several other DEGs were included in amino acid metabolism pathways such as valine, leucine and isoleucine degradation (*HADHB*, *ACADM*), arginine biosynthesis (*ASS1*) and histidine metabolism (*CARNMT1*). In addition, two signaling pathways, the oxytocin and prolactin signaling pathway, might be associated with reproductive traits.

### Integrative analysis of whole genome sequencing and RNA-seq

**Figure 5:**
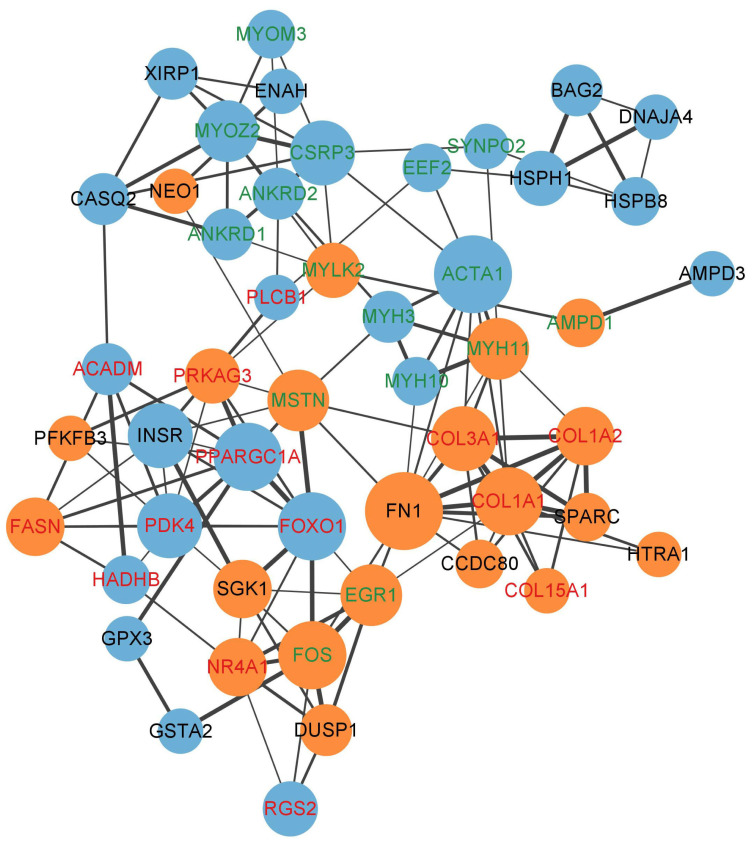
Protein-protein interaction analysis showing the relationships of the significant DEGs. The size of the nodes represents the p-value of the DEGs, with a large node representing a low p-value, and a small node representing a high p-value. Orange nodes indicates up-regulated genes, and blue nodes indicates down-regulated genes. A red label represents DEGs related to meat quality, and a green label represents DEGs involved in the muscle development process. More lines between two genes represents more possible interactions. DEGs, differentially expressed genes.

The candidate genes of JG under positive selection and the DEGs were subjected to cross-comparisons, and five genes (*ABHD2*, *FN1*, *PGM2L1*, *PRKAG3*, *RAVER2*) that overlapped between the two gene sets were identified (Figure 4D). The PPI network analysis revealed the relationships of these genes. As shown in Figure 5, an orange node indicates upregulated genes, and a blue node indicates downregulated genes. Additionally, a red label represents DEGs related to meat quality, and a green label represents DEGs involved in the muscle development process. Thicker line between two genes represents more possible interactions and larger size of node represents more genes associated with it. The network analysis results indicated that genes associated with meat quality presented a close relationship, as did the genes involved in the muscle development process. Two of the five overlapping genes (*FN1* and *PRKAG3*) corresponded to the critical node in the network.

## DISCUSSION

The meat quality has gradually become a focus of attention in the consumption process. While, high quality represents good taste, unique flavor, and high nutritional value [11]. Fatty acid and amino acid not only constitute important nutritional components in lamb meat, but also affect the flavor of lamb meat as volatile compounds and precursors. Therefore, fatty acids and amino acids metabolism was considered as important biological processes that affect meat quality [9]. In order to reveal the metabolism of meat quality and muscle development of JG, we performed multiomics analyses based on which JG was compared with BG, which is the most productive meat goat breed and has thus been introduced to most regions of the world [28]. First, WGS was carried out to screen the selective signatures associated with meat quality and muscle development. Then, transcriptomic analysis of the *longissimus thoracis* identified DEGs between the two goat breeds. The results revealed 442 candidate genes under positive selection were identified in JG and 139 genes were found to be differentially expressed. The GO enrichment and KEGG pathway analyses for the two sets of genes revealed multiple candidate genes and signaling pathways related to meat quality and muscle development, respectively. Although only five overlapped between the two sets of genes, numbers of pathways related to lipid and amino acid metabolism were identified in both analyses. These pathways included the FoxO signaling, Apelin signaling, and valine, leucine and isoleucine degradation pathways. Among the five genes, *ABHD2* and *PRKAG3* participate in multiple signaling pathways related to lipid metabolism. *ABHD2* belongs to the mammalian α β hydrolase domain (ABHD)-containing proteins, and human ABHD2 was identified as a novel triacylglycerol (TAG) lipase and ester hydrolase [29]. The family member *ABHD5* has been found to be regulated by Evi1 and C/EBPα and could be used as a potential marker in marker-assisted selection for the improvement of the Qinchuan cattle breed for carcass quality traits [30]. However, reports about the involvement of *ABHD2* in meat quality traits of goat were not found. *PRKAG3* encodes the γ3 subunit of the 5’AMP-activated protein kinase (AMPK) that controls cellular energy homeostasis in response to environmental or nutritional stress. Variation in this gene has been found to be associated with meat quality traits by affecting meat pH and tenderness in cattle [31] and sheep [32]. But similar studies have not been reported in goat yet.

In the transcriptomic analysis, more genes related to lipid metabolism and muscle development were identified. They were not found in the selection signature analysis, possibly because the mutations occurred in other selection regions caused their differential expression at the transcriptional level through regulatory pathways. Among them, four collagen genes (*COL1A1*, *COL1A2*, *COL3A1*, *COL15A1*) were upregulated in JG. The mechanism by which collagen affects meat quality is complex. First, increases in muscle collagen contents and the decreases in muscle collagen solubility can improve the water holding capacity of muscle but also reduce tenderness [33]. On the other hand, collagen can promote adipose tissue accrual via mechanisms that determine adipocyte number and thereby affect fat deposition [34], significantly improving the water holding capacity of muscle without affecting tenderness. The other two downregulated genes (*ACADM* and *HADHB*) related to both fatty acid metabolism (fatty acid degradation) and amino acid metabolism (valine, leucine and isoleucine degradation) might be related to the outstanding meat quality and flavor of JG. *HADHB*, one of the rate-limiting enzymes for the beta-oxidation of fatty acids, was identified as a candidate biomarker for intramuscular fat deposition in chickens [35].

For muscle development, a set of candidate genes and signaling pathways was identified by multiomics analyses. In particular, the upregulated *MSTN* gene in JG is assumed to be closely related to its low slaughter rate. Numerous studies on the genetic mechanism and modification of this gene in goats have recently been reported [36]. In addition, *PPARGC1A* and *MSTN* were enriched under the GO term skeletal muscle atrophy. *PPARGC1A*, encoding peroxisome proliferator-activated receptor gamma coactivator 1 alpha, has been reported to participate in skeletal muscle development and fatty acid oxidation by regulating the number and respiration of mitochondria [37]. Studies in chickens have shown that this gene is highly expressed in slow-twitch myofibers and can promote intramuscular fatty acid oxidation, drive the transformation of fast-twitch to slow-twitch myofibers, and increase chicken skeletal muscle mass [13]. Similarly, *ANKRD2* and *MSTN* were enriched under the GO term negative regulation of myoblast differentiation. *ANKRD2* is a member of the muscle ankyrin repeat protein family. As a striated muscle signaling protein and transcriptional regulator, it participates in myogenesis, myogenic differentiation, muscle adaptation and stress responses. It is preferentially expressed in slow, oxidative fibers of mammalian skeletal muscle [38]. *ANKRD2* is not necessary for life, nor is it the direct cause of any muscular disorder, and multiple lines of evidence suggest that an anomalous expression level of *ANKRD2* might contribute to a muscular phenotype [39]. In this research, *PPARGC1A* and *ANKRD2* were both found to be downregulated in JG, which might account for its low meat production performance.

In conclusion, the comparative genomic and transcriptomic analysis of JG and BG revealed the mechanisms underlying the meat quality and meat productive performance of goats. A number of candidate genes associated with lipid metabolism, amino acid metabolism and muscle development were revealed. In particular, several identified genes have been rarely reported in goats, such as *ABHD2*, *ACADM*, and *HADHB*, which are associated with lipid metabolism, and *PPARGC1A* and *ANKRD2*, which are associated with muscle development. These results provide valuable information for future breeding in goats.

## Data Availability

The datasets generated during the current study have been deposited to the NCBI Sequence Read Archive, under the BioProject accession number PRJNA981773.

## SUPPLEMENTARY MATERIAL

**Supplementary Table S1.** Overview of whole-genome sequencing and alignments statistics

**Supplementary Table S2.** Number of SNPs and INDELs for each individual

**Supplementary Table S3.** A summary of the RNA-seq reads alignment to the goat reference genome

**Supplementary Table S4.** A summary of the RNA-seq reads alignment tothereference genes

**Supplementary Table S5.** Gene expression and annotation by RPKM

**Supplementary Table S6.** Gene listfor selective sweep results

**Supplementary Figure S1.** Phylogenetic tree of 22 individuals. **Supplementary Figure S2.** Principal component analysis of 22 individuals.
