# Supplementary material for: Multiomics analyses of Jining Grey goat and Boer goat reveal genomic regions associated with fatty acid and amino acid metabolism and muscle development
Source: Anim Biosci. 2023 Nov 2;37(6):982–92. doi: 10.5713/ab.23.0316 (PMC11065957; doi:10.5713/ab.23.0316)
Supplement: Supplementary file 1 [file ab-23-0316-Supplementary-Table-1.pdf]

Supplementary Table 1. Overview of whole-genome sequencing and alignments statistics.

Sample	Clean	Mapped-reads	Mapped-rate(%)	Coverage(%)	Aver-dep
JG1	212154760	210627245	99.28	99.64	11.32
JG2	226946200	224971768	99.13	91.57	11.63
JG3	192380396	190918304	99.24	99.61	10.28
JG4	233594620	231819300	99.24	99.64	12.43
JG5	296891342	294664656	99.25	99.68	15.79
JG6	248298734	246436493	99.25	99.64	13.3
JG7	348800754	346010347	99.2	99.66	18.68
JG8	329530888	326927593	99.21	99.67	17.51
JG9	320398524	317931455	99.23	99.67	17.18
JG10	302625136	300022559	99.14	99.65	16.15
JG11	327230054	324088645	99.04	99.67	17.51
JG12	317221238	314715190	99.21	99.66	16.95
AB1	324533950	322554292	99.39	99.33	11.65
AB2	347702714	345164484	99.27	99.41	12.36
AB3	359185776	356599638	99.28	99.58	12.92
AB4	354542152	352131265	99.32	99.69	12.75
AB5	314327826	312253262	99.34	99.57	11.36
AB6	328649254	326513033	99.35	99.58	11.83
KB1	311187556	308791411	99.23	99.24	11.23
KB2	315872564	313471932	99.24	99.21	11.41
KB3	297228620	295148019	99.3	99.12	10.73
KB4	305659424	303519808	99.3	99.15	11.02
