# Supplementary material for: Multiomics analyses of Jining Grey goat and Boer goat reveal genomic regions associated with fatty acid and amino acid metabolism and muscle development
Source: Anim Biosci. 2023 Nov 2;37(6):982–92. doi: 10.5713/ab.23.0316 (PMC11065957; doi:10.5713/ab.23.0316)
Supplement: Supplementary file 2 [file ab-23-0316-Supplementary-Table-2.pdf]

Supplementary Table 2. Number of SNPs and INDELs for each individual.

type	all-snp	hom	het	all-indel	deletion	insertion
JG1	6556444	2113534	4442910	502840	263292	239548
JG2	5772985	1706319	4066666	387882	204531	183351
JG3	5779792	2568730	3211062	430653	223558	207095
JG4	6035791	3041208	2994583	465530	239807	225723
JG5	7412160	2235577	5176583	581812	303821	277991
JG6	6345949	2962465	3383484	494557	255656	238901
JG7	7396968	2453386	4943582	584951	305662	279289
JG8	7441230	2362665	5078565	586165	305834	280331
JG9	7358013	2417592	4940421	580882	303023	277859
JG10	7353474	2428408	4925066	575405	300722	274683
JG11	7236639	2584615	4652024	573752	299037	274715
JG12	7342223	2441139	4901084	580153	302442	277711
AB1	3916340	1006881	2909459	277201	148658	128543
AB2	4112339	1024195	3088144	285433	154494	130939
AB3	4402231	1701590	2700641	330238	172394	157844
AB4	5701796	1704028	3997768	425953	224516	201437
AB5	4913319	1526588	3386731	365833	193179	172654
AB6	4767417	1370407	3397010	351101	185397	165704
KB1	6972018	1592623	5379395	504117	269135	234982
KB2	6986585	1605173	5381412	506284	270293	235991
KB3	5919070	2257464	3661606	434711	227329	207382
KB4	5965868	2262115	3703753	438736	229059	209677
