# Supplementary material for: Multiomics analyses of Jining Grey goat and Boer goat reveal genomic regions associated with fatty acid and amino acid metabolism and muscle development
Source: Anim Biosci. 2023 Nov 2;37(6):982–92. doi: 10.5713/ab.23.0316 (PMC11065957; doi:10.5713/ab.23.0316)
Supplement: Supplementary file 3 [file ab-23-0316-Supplementary-Table-3.pdf]

Supplementary Table 3. A summary of the RNA-seq reads alignment to the goat reference genome.

Sample	JG	BG
Total Reads	3711416(100%)	3648953(100%)
Total BasePairs	181859384(100%)	178798697(100%)
Total Mapped Reads	2863343(77%)	2823329(77%)
perfect match	2049528(55%)	1978574(54%)
<=3bp mismatch	813815(22%)	844755(23%)
unique match	2361451(64%)	2321499(64%)
multi-position match	501892(14%)	501830(14%)
Total Unmapped Reads	848073(23%)	825624(23%)
