# Supplementary material for: Multiomics analyses of Jining Grey goat and Boer goat reveal genomic regions associated with fatty acid and amino acid metabolism and muscle development
Source: Anim Biosci. 2023 Nov 2;37(6):982–92. doi: 10.5713/ab.23.0316 (PMC11065957; doi:10.5713/ab.23.0316)
Supplement: Supplementary file 4 [file ab-23-0316-Supplementary-Table-4.pdf]

Supplementary Table 4. A summary of the RNA-seq reads alignment to the reference genes.

Sample	JG	BG
Total Reads	3711416(100%)	3648953(100%)
Total BasePairs	181859384(100%)	178798697(100%)
Total Mapped Reads	1521709(41%)	1452037(40%)
perfect match	1293803(35%)	1232824(34%)
<=2bp mismatch	227906(6%)	219213(6%)
unique match	1384464(37%)	1340089(37%)
multi-position match	137245(4%)	111948(3%)
Total Unmapped Reads	2189707(59%)	2196916(60%)
