# Supplementary material for: Multiomics analyses of Jining Grey goat and Boer goat reveal genomic regions associated with fatty acid and amino acid metabolism and muscle development
Source: Anim Biosci. 2023 Nov 2;37(6):982–92. doi: 10.5713/ab.23.0316 (PMC11065957; doi:10.5713/ab.23.0316)
Supplement: Supplementary file 5 [file ab-23-0316-Supplementary-Table-5.pdf]

Supplementary Table 5. Gene expression and annotation by RPKM.

RPKM	Gene number of JG (%)	Gene number of BG (%)
0-100	11519 (93.57%)	11289 (93.30%)
100-1000	715 (5.81%)	727 (6.01%)
>10,00	77 (0.63%)	84 (0.69%)
Total	12311	12100
