# Supplementary material for: Multiomics analyses of Jining Grey goat and Boer goat reveal genomic regions associated with fatty acid and amino acid metabolism and muscle development
Source: Anim Biosci. 2023 Nov 2;37(6):982–92. doi: 10.5713/ab.23.0316 (PMC11065957; doi:10.5713/ab.23.0316)
Supplement: Supplementary file 6 [file ab-23-0316-Supplementary-Table-6.pdf]

Supplementary Table 6. Gene list for selective sweep results.

type	count	gene list
$Z(H_P)_{JG}$ < -1.498	1563	U6, RNaseP_nuc, U4, GP5, SYNJ1, 7SK, DIP2A, P2RY14, P2RY13, SCAF4, KALRN, GPR171, DNAJC28, CPN2, PRMT2, NRROS, MCF2L2, NSUN3, TMEM50B, CMSS1, TRIM59, PHC3, PLSCR5, STAG1, DOP1B, ARL13B, DZIP1L, CD86, SHOX2, CRYBG3, LRRC15, GRAMD1C, MAP6D1, A4GNT, CRYZL1, MSL2, GART, TBC1D23, ATP6V1A, DONSON, PAXBP1, CBR3, P2RY12, KAT2B, RYK, KY, NCEH1, ABCC5, PLCH1, ATP13A3, IFT80, SLC51A, UPK1B, PLSCR1, DYRK1A, EAF2, GPR87, TOPBP1, FBXO45, BCHE, DBR1, CASR, KPNA4, SCARNA14, U2, PSMB11, TOX4, SNORD16, LRP10, SNORD18, 5S_rRNA, PLCB2, LTBP2, MINDY2, U1, TCF12, PROX2, IRF2BPL, MMP14, ANKRD63, SNAPC5, MYO9A, ZFYVE1, DTWD1, REM2, CGNL1, SLC24A5, USP8, CTXN2, MRPL52, LHFPL2, SIMC1, CCNB2, PTPN21, FGF7, GRAMD2A, PKM, RPS6KL1, KCNK10, HEATR4, LCTL, NUMB, DLGAP5, BMF, WDHD1, EML5, AP3B1, GCH1, NPC2, GPR137C, USP3, SLC12A1, TIMM9, UBR1, DCAF5, CERT1, PGF, OXA1L, ADPGK, FBXL22, AREL1, RNF111, ZNF106, DENND4A, GLDN, HOMER1, STYX, RPGRIP1, DHRS7, LIPC, RABEPK, GAPVD1, BIRC6, AIF1L, ODF2, chi-mir-181b, SUPT7L, FNDC4, ZC3H8, YWHAQ, PNO1, PMPCA, ROCK2, IFT172, PEX13, SPTAN1, ADAM17, RBKS, RPS27A, EHBP1, PTRHD1, GGCX, C2orf74, ARPC5L, ADGRD2, GARNL3, CIB4, E2F6, ATAD2B, VRK2, CYP26B1, TTC27, PLPP7, OTOF, PKN3, SDC1, RALGPS1, PBX3, REL, TMEM150A, TPRKB, PUM2, WDR38, OLFML2A, EDAR, SLC30A6, SFTPB, RABGAP1, GLE1, LAMC3, ADCY3, DUSP11, NUP214, YIPF4, C2orf68, KIDINS220, MEIS1, UGGT2, SNORA70, chi-mir-16a, MIR15A, ZMYM2, OXGR1, IL17D, SPRYD7, DIS3, PARP4, SAP18, INTS6, SUCLA2, DOCK9, SLC15A1, MZT1, LPAR6, TDRD3, MPHOSPH8, IFT88, EPSTI1, NBEA, ADARB2, Y_RNA, U5, SRC, TAF3, SNORA71, DDRGK1, BMP7, EYA2, MLLT10, ITGB1, MASTL, PPP1R3D, MMP24, ITCH, SYCP2, LARP4B, ZNF438, WFDC11, BPIFB4, UBE2C, TTLL9, SLC4A11, MYLK2, MAP3K8, DYNLRB1, ATP5F1C, ZSWIM3, BCAS4, VIM, RNF24, DNAAF9, MAP1LC3A, DNTTIP1, PLXDC2, ARMC3, ITIH2, TPX2, IDI1, WFDC10A, DIP2C, PARD6B, ADIG, TP53INP2, NNAT, SNX21, RALGAPB, ADCK5,

		LY6E, FABP4, PHF20L1, HSF1, CRH, NCOA2, PKHD1L1, STAU2, CYP11B1, BPNT2, E2F5, DECR1, FABP9, INTS8, SLC52A1, ELOC, TMEM67, SLC26A7, KHDRBS3, PDE7A, LRRC69, SCX, POP1, MAF1, PMP2, CYC1, CA13, MROH1, LRRCC1, DPY19L4, EMC2, PDP1, EYA1, MATN2, FABP12, HGH1, TG, OSGIN2, OR52W1, MIR125B1, DNHD1, KCNA4, BCL9L, CD3D, ALKBH8, OR56B4, CFAP300, TIMM8B, CEP126, C2CD3, ART1, DPAGT1, BIRC3, H2AX, LIN7C, CEP57, CCDC153, PIH1D2, TEAD1, YAP1, MPPED2, BIRC2, ILK, HYOU1, STIM1, AASDHPPT, SYT9, PHOX2A, FAR1, RRM1, RDX, NCAPD3, IL18, P4HA3, MRE11, WNT11, ADM, ARHGAP42, C11orf42, SLC37A4, PGM2L1, DDX6, FAM168A, IFT46, CNGA4, CLPB, PGAP2, LGR4, MIR3120, SRM, CHML, TNFRSF18, FH, NPHP4, FNDC10, ADORA1, TNFRSF9, DNM3, MRPL20, TNFRSF25, ELF3, TIMM17A, MAD2L2, NOL9, ZBTB17, CASP9, ICMT, SLC35E2B, UBE2T, CFAP74, PHLDA3, CASZ1, FBXO2, SDF4, RRP15, TMEM240, UBE2J2, HLX, HES2, CHD5, DVL1, GPR153, IPO9, AURKAIP1, C1orf112, DISP1, TNNI1, UTS2, LZIC, BTG2, KLHL20, PLD5, MASP2, RGL1, ACAP3, SMG7, PANK4, CCNL2, MORN1, TNFRSF4, MTOR, EXOSC10, PLEKHG5, ZBTB37, ANKRD65, C1orf159, C1QTNF12, CSRP1, TNNT2, RNPEP, MTARC2, METTL13, HES5, TGFB2, QSOX1, INTS11, MXRA8, NADK, XPR1, CEP350, SYT2, RPL22, NPL, ACOT7, DARS2, RERE, TMEM52, RABGAP1L, MMEL1, PACC1, AGMAT, PITPNB, KLHL2, PHETA1, RNF34, SPATA5, ATXN2, SDSL, SUDS3, TTC28, JADE1, BRI3BP, POP5, AACS, BRAP, PGAM5, COQ5, TMEM131L, FBXW8, RAB33B, MN1, NAA15, RNFT2, P2RX2, LARP1B, NOCT, MSI1, GAB1, PXMP2, PLBD2, SRSF9, TLR2, FBRSL1, chi-mir-99b, MIRLET7E, SNORD68, MMP15, TIMM50, INAFM1, C5AR2, SNORD35B, SNORD35A, HSD11B2, SNORD34, ZNF432, KIAA0513, EXOSC6, MED25, SETD6, CCDC9, CEBPG, SNORD33, PLA2G15, MIR125A, TOX3, PRMT7, SPACA4, CDK10, ZNF473, PTOV1, ZFPM1, SLC7A6, SLC9A5, RCN3, LRRC36, CATSPERG, SPACA6, TRIM28, LRP3, DPEP1, FLT3LG, HAS3, UBA2, RYR1, FUT2, CNFN, CCDC113, BCL2L12, PRMT1, PLEKHG4, AARS1, SPG7, TANGO6, C5AR1, PLEKHG2, ATF5, TRPM4, COG4, ZNF276, ZFHX3, HIF3A, RPS5, SPINT2, SCAF1, ERF, PAFAH1B3, ZNF613, FAM98C, TBC1D17, DEDD2, LIPE, ZNF614, GINS3, KCNK6, NDRG4, CPNE7, ALDH16A1, LYPD4, RNF225, NFATC3, UTP4, MED29,

		DUS2, SULT2B1, DKKL1, ZFP36, PRRG2, CD37, SUPT5H, ST3GAL2, CCDC61, SPHK2, SNTB2, DHX34, PPP5C, FCGRT, AP2A1, FGF21, CPT1C, WTIP, PSMD8, SAE1, CYB5B, ZNF446, SAMD4B, KASH5, RPS11, SPRED3, TNFSF12, SNORD42, SCARNA16, TANC2, MIR152, PSMD3, SNORA48, HEXIM1, SNORD10, CASC3, HEXIM2, FGF11, MRC2, PRR15L, CFAP52, NMT1, SNORA67, B3GNTL1, TNK1, PEMT, TP53I13, DOC2B, NCOR1, SNORD4A, YPEL2, AATF, ELP5, OMG, CAMTA2, THRA, PLD6, INCA1, COPS3, MYADML2, CSNK1D, EVI2B, ACE, SOCS7, TLK2, SGSM2, MSL1, CRYBA1, MRPL45, CHRNB1, TMEM102, RAPGEFL1, USP43, MYO1D, CSF3, NEK8, RAD51C, GSDMA, STXBP4, CDK12, MAFG, WRAP53, LRRC75A, SPEM2, SIRT7, SRSF2, TAOK1, NOTUM, ACAP1, SRR, DNAH2, FASN, COPRS, SNORD4B, ACBD4, TRAF4, TNFSF13, FAAP100, MPDU1, DUS1L, SHBG, FOXN1, SLC2A4, ZNF232, NLGN2, CRLF3, LHX1, ATP1B2, CD68, RASD1, SPEM1, ZSWIM7, PAFAH1B1, TMEM95, C17orf75, SUPT6H, ACADVL, ST6GALNAC1, PEX12, ANKRD13B, ZNF750, SPAG7, RFNG, COPZ2, PPM1E, PYCR1, SLFN14, DCXR, APPBP2, POLR2A, FLCN, EFNB3, SNORA73, METTL21A, LDLRAP1, RAB3GAP1, KLHL23, ZDHHC18, USP37, PLEKHM3, chi-mir-10b, HOXD13, RNF25, NIFK, ARID1A, C1QC, HOXD3, HOXD10, PDK1, MYO3B, DHDDS, HECW2, ITGA6, ACVR2A, HOXD8, TRIP12, SLC35F5, UBR3, HOXD12, NHEJ1, RPS6KA1, CFAP65, FEV, GTF3C3, IHH, WDR75, CYFIP1, PHACTR4, C1QB, HOXD1, ANKRD44, GPD2, PGAP1, PKP4, MARCHF7, HOXD4, MED18, DNER, CRYBA2, NPPC, FN1, FLACC1, FTCDNL1, CLASP1, EPHB2, TRNAU1AP, HOXD9, EPHA8, PRKAG3, CSRNP3, ALS2, AAMP, EPC2, PIGV, TMEFF2, PID1, TNP1, TUBGCP5, TMEM237, OCA2, TMBIM1, PINK1, KYNU, TMEM50A, HOXD11, TRAK2, MACO1, RCC1, DIMT1, IL6ST, TLX3, FGF18, CD180, FGF10, GABRP, CDH12, SPDL1, NUP155, PDE4D, FYB1, SLC9A3, CPLANE1, RICTOR, CCDC127, ADAMTS6, CDH10, FANCI, SNORD107, SNORA28, HHIPL1, IMP3, NUBPL, KLF13, TICRR, MIR9, MIPOL1, FAH, HDGFL3, ZNF592, TARS3, PPP4R4, ALPK3, BRMS1L, TM2D3, PCSK6, OTUD7A, EML1, MAN2C1, UNC79, PAX9, SNRPN, SNUPN, PTPN9, MARK3, MESP2, WDR93, INSM2, BNC1, APBA2, SIN3A, PSMA6, MKRN3, FAN1, TRMT61A, EIF5, CSPG4, UBE2Q2, MPHOSPH10, COA8,

		GABRG3, FBXO22, CHSY1, CYP46A1, SRP54, FAM177A1, ERC2, RRP9, DCAF1, OXTR, RAD18, PLXND1, RBMS3, MIR26A, C3orf62, MRPS25, FAM3D, DENND6A, SFMBT1, NICN1, CFAP20DC, IQCF1, SHQ1, VOPP1, CCDC71, PCBP4, CFAP100, STT3B, RHOA, GLT8D1, CCDC12, C3orf84, DBNL, CTDSPL, OXSR1, NEK4, USP4, MYD88, GPX1, ARF4, PBRM1, SETD2, THOC7, TASOR, LHFPL4, SRGAP3, SUCLG2, ITGA9, DLEC1, VILL, SSUH2, KLF15, DNAH12, ABHD14B, KIF9, NT5DC2, IQCF5, PSMD6, ZNF621, RYBP, SLC22A13, EGFL8, U7, CPNE5, SMIM13, ZNF391, VARS2, H2BC1, ZNF165, CYP21A1, E2F3, CILK1, EDN1, ZSCAN16, ZKSCAN4, SLC17A4, ELOVL5, RNF5, ZSCAN26, PGBD1, TRAM2, SCGN, ZKSCAN8, GSTA4, HIVEP1, PXT1, GCM1, SYCP2L, TULP1, TEAD3, ILRUN, C6orf136, DDR1, MRPS18B, PKHD1, GTF2H4, ATAT1, KHDRBS2, BNIP5, TUBB, INO80C, ZNF24, NOL4, ZNF521, ADCYAP1, LDLRAD4, B4GALT6, RNMT, SNORD36, TRAPPC8, PTPRM, RELCH, MC5R, PHLPP1, KATNAL2, PARD6G, TNFRSF11A, SNORD14, CRAMP1, MIR25, BAIAP2L1, HS3ST2, HBM, NUDT16L1, RAB11FIP3, RHBDL1, TMEM204, chi-mir-106b, MGRN1, DNASE1L2, POLR2J2, chi-mir-93, RFC2, FAM234A, PRR35, JPT2, WDR90, PIGQ, WFIKKN1, STUB1, HIRIP3, HBQ1, GNPTG, ITGAL, PRR14, TELO2, DOC2A, TBX6, HAGHL, CALN1, VWA3A, DTX2, KCTD7, BFAR, GDPD3, RRN3, PDGFA, CRCP, TLCD3B, BAIAP3, MAPK3, NHLRC4, UQCRC2, MCRIP2, RHOT2, UBE2I, CACNA1H, ZNF3, GPC2, MOSMO, PDIA2, ZNF771, MRTFB, CDIPT, E4F1, LYRM1, SEC14L5, ALKBH4, TPST1, CAPN15, RNF216, MAPK8IP3, ANTKMT, TRAPPC14, CCNF, YPEL3, REXO5, RAB40C, DECR2, KCTD13, NME4, ZCWPW1, UBN1, NUBP2, ACSM3, SEZ6L2, HBAI, TECPR1, ARHGDIG, GAL3ST4, HMOX2, UPK3B, PRKAR1B, METRN, MCM7, TAF6, SHTN1, HSPA12A, POLL, PITX3, SNORD22, HPSE2, CUEDC2, RGS10, ADAM12, ATE1, LDB1, OGA, SEC31B, FGFBP3, MCMBP, BBIP1, PSD, FGF8, CPEB3, FBXW4, PIK3AP1, ARMH3, C10orf82, KCNIP2, IKZF5, PPP1R3B, FGFR1, ZDHHC2, UFSP2, THAP1, CENPU, LSM1, GOT1L1, GSR, MFHAS1, RNF170, STAR, MICU3, PCM1, CSMD1, TM2D2, ACSL1, LRP2BP, CHRNA6, PDLIM3, MMRN2, ANK3, MYOZ1, DNAJB12, MIR1296, COMTD1, FAM89A, GDF10, CAMK2G, PGBD5, RBP3, DUSP29, USP54, GDF2, FAM13C, EGR2, MICU1, JMJD1C, HEATR1,

		DUSP13, SNORD5, SCYL1, SNORA25, SNORA8, FADD, SIPA1, SNORD6, FRMD8, CHORDC1, FAM89B, EHBP1L1, PCNX3, GAB2, NARS2, ADAMTS15, ZNRD2, ANO1, VSTM5, TAF1D, ST14, ZYG11B, PLK3, LENEP, GJB3, ELAVL4, SNORD45A, EFNA1, RAVER2, SCP2, GJA4, ADAM15, SNORA62, PASK, PDE4B, PROK1, MACF1, EFNA4, RLF, C1orf146, WNT2B, PKN2, LRRC8D, GJB4, TRIM46, GJB5, TMEM125, DDX20, CLCA1, S100A1, STXBP3, STRIP1, CACHD1, CKS1B, MTX1, MAN1A2, TIE1, BTBD19, MAGI3, RUSC1, CD53, ZMPSTE24, FNBP1L, RNF11, BTBD8, DNAJB4, PODN, ST6GALNAC5, NEXN, EPHX4, ZNF326, DAP3, HCN3, RAP1A, MIER1, FAM102B, CTTNBP2NL, ATF6, FNDC7, SNAPIN, SHISAL2A, RPAP2, FDPS, RNPC3, SLC50A1, TMCO2, DR1, LRIG2, SLC6A17, UBAP2L, TFAP2E, CLCA4, DCST1, MTERF4, FGGY, HDLBP, SLC27A3, AHCYL1, NPR1, GIPC2, ABCD3, USP40, TLCD4, PRKACB, CHTOP, ADAMTSL4, C1orf185, PRPF38A, PTBP2, MPL, HOOK1, SZT2, FLAD1, CREB3L4, INTS3, ZBTB7B, PALMD, SMAP2, MSH4, NCDN, POU2F1, RBM15, UROD, ZMYM4, CSF1, ROR1, ZFYVE9, MSTO1, U3, FBXL13, VSTM2A, PRPS1L1, ZNRF2, FAM221A, SP8, GPR141, CNOT4, MTURN, KMT2C, MTERF1, PHTF2, MNX1, PTPN12, LMBR1, SRI, ZNF746, RELN, WASL, LAMB4, OPN1SW, CFTR, IMPDH1, BRAF, HECW1, STK31, PALS2, AEBP1, DNAJB9, DNAJC2, ZNF777, PLEKHA8, SRPK2, TOMM7, FOXP2, SLC26A5, NAPEPLD, LAMB1, TES, CCDC146, AGAP2, ZBTB39, SNORA2C, MIRLET7A, KICS2, CLEC12A, SCARNA11, ERC1, ERGIC2, LPAR5, TMTC3, PTHLH, MAP3K12, MIRLET7B, NCAPH2, CYP27B1, SLC38A2, CELA1, PUS7L, ACRBP, CLEC12B, ADCY6, MYO1A, DDIT3, RPAP3, WIF1, ZNF384, C1QL4, PPM1H, SARNP, ARHGAP9, PANX2, R3HDM2, MLF2, BCDIN3D, LRP6, GRAP2, FMNL3, NEMP1, METTL1, DNAI7, IQSEC3, SCAF11, AMN1, AVIL, CSRNP2, GALNT6, PIANP, WASHC4, TRABD, LGR5, MOV10L1, NOP2, LLPH, FAIM2, IFFO1, MCRS1, DENND6B, NPFF, GXYLT1, DENND5B, MMP19, CDK4, ELK3, CHD4, SELENOO, DCTN2, MIOX, TFCP2, THAP2, TMTC1, TIMP3, TMEM19, PARPBP, CLEC1A, TWF1, ITGA7, PLBD1, CCNT1, ING4, ADM2, STAT6, YAF2, KANSL2, PYM1, SOX5, TAC3, LIMA1, PPARA, PLEKHG7, KRAS, B4GALNT1, NCKAP5L, C4orf48, PCDH7, AFF1, NSD2, CRACD, POLR2B, ARL9, NCAPG, TBCK, STPG2, NSUN7,

		TBC1D14, NAT8L, SOD3, KIAA0232, PRDM8, SLC9B1, STX18, ADGRL3, DNAJB14, MANBA, SRP72, GRK4, HPGDS, KIT, CPEB2, ARHGAP24, ENOPH1, ADD1, LAMTOR3, THEGL, FGF5, LEF1, UBE2D3, TMEM59L, MAN2B1, SNORD63, MED16, LYSMD3, SNORD95, SNORD96, GNA15, GDF9, CTXN1, PLPPR2, ELL2, PLPPR3, SLC25A2, CDKL3, NMRK2, KLHL26, ARAP3, PJA2, RFXANK, GAMT, KLF16, SWSAP1, GFRA3, STING1, RAB24, PROB1, PFDN1, SLC23A1, GNA11, FBN3, MXD3, TCF3, MEX3D, PCDH1, PRKCSH, ZNF555, THOP1, POLR2E, GIN1, ELAVL1, NME5, AFF4, PIAS4, C19orf25, RACK1, SPATA24, FAM13B, ZNF300, ABHD17A, PCSK4, HARS1, MAU2, TLE6, SRFBP1, CNN1, HSPA9, R3HDM4, FCHSD1, CRTC1, WDR83OS, BRD8, ZNF554, ZCCHC10, SPOCK1, TRIM52, SAR1B, FBXL17, DNAJC18, PRSS57, MZB1, NCAN, HAPLN1, PPP2CA, HDAC3, UBA52, MBD3, PCDH12, CDC25C, GABRB2, TNIP1, SNAP47, CCDC159, CDC23, DIAPH1, CHD1, BTBD2, TRIM7, REX1BD, DND1, ATCAY, TMEM259, CDKN2AIPNL, KXD1, NANS, SCARNA8, DNAJB5, TDRD7, FOXE1, PPP2R2A, ZNF462, MIRLET7F, MIRLET7D, MIRLET7A1, SPINK4, RPS6, RFX3, AOPEP, NUDT2, GALNT12, PLIN2, ANXA10, SAXO1, MFSD14B, MYT1L, PXDN, UBAP1, XPO7, CER1, PTPRD, HABP4, SFTPC, PHF24, DMTN, TLE4, NFX1, TMOD1, VPS13A, FHIP2B, ZDHHC21, RUSC2, CFAP95, TGFBR1, BMP1, HAUS6, NPM2, BNC2, FGF17, COL15A1, CHMP5, DPYSL2, ENPP3, SNORA33, SNX3, SNORD101, PPIL6, CLDN20, SNORD100, SNORD50B, BCKDHB, CNR1, COL10A1, GABRR1, FYN, TTK, FAXC, SCML4, COQ3, RARS2, TBX18, TSPYL1, USP45, SRSF12, RPS12, GINM1, MICAL1, TIAM2, PNISR, SNX14, SESN1, OSTM1, RNGTT, MMS22L, EPHA7, AKIRIN2, SEC63, TRAPPC3L, CD164, ARG1, CD109, BACH2, GJB7, CEP43, HDAC2, ZBTB24, SYNCRIP, GRM1, PCMT1, PDE7B
$Z(F_{ST}) > 1.773$	1101	U6, RIPPLY3, 5S_rRNA, SLITRK3, ADAMTS1, YBEY, CLDN17, MIR802, PLD1, SI, KRTAP24-1, IQCG, GTF2E1, NR1I2, TMEM50B, CMSS1, STAG1, AMOTL2, DZIP1L, RAB6B, COL8A1, RUBCN, TTC3, SLC7A14, PDE9A, A4GNT, TIAM1, PLOD2, ADAMTS5, AGPAT3, SLCO2A1, PKNOX1, ANAPC13, RBP1, ASTE1, STXBP5L, COPB2, RYK, SPATA16, KY, PCOLCE2, SIM2, CYYR1, ACAD11, PLSCR1, DYRK1A, EAF2, NMNAT3, APP, TOPBP1, CLDN8, DBR1, RBP2, RHOV, U2, ACTC1, NRDE2, FOXN3,

		SPG21, LTBP2, TMEM87A, PLA2G4F, PPP1R14D, CCDC177, RASL12, DTWD1, CGNL1, LARP6, PTPN21, VPS39, MTFMT, FGF7, DNAJC17, MAP3K9, KCNK10, HEATR4, GJD2, NUMB, UACA, EFCAB11, EML5, PIGH, NPC2, SLC10A1, GPR137C, CERT1, AREL1, TMEM229B, RMDN3, TRDC, DPF3, SERINC5, AQR, PLA2G4D, PTGER2, PLA2G4E, KIF3C, SNORA70, AFTPH, SMC6, CNGA3, CNNM4, CNNM3, ANAPC1, UGP2, TPRKB, C2orf73, ASXL2, INPP4A, DUSP11, MBOAT2, CRB2, LGALSL, KCTD4, PDS5B, LCP1, KBTBD6, KBTBD7, chi-mir-16a, MIR15A, MTRF1, RUBCNL, TSC22D1, GPR183, SPRYD7, HTR2A, FRY, SOX21, BRCA2, KLHL1, ENOX1, ELF1, GPR18, EPSTI1, TGDS, RPN2, CACNB2, PTER, ADARB2, TMEM236, STAM, EPC1, ODAD2, DNMT3B, CAMK1D, PET117, SNORA71, APCDD1L, DDRGK1, SCRT2, BMP7, RIN2, ROMO1, DIDO1, AVP, PRND, SLC24A3, EYA2, MLLT10, CENPB, CTSZ, ADAM33, FASTKD5, UBOX5, MMP24, C20orf27, ITCH, LARP4B, ZNF438, EFCAB8, PRNP, GATA5, MSRB2, SRXN1, ABI1, CHMP4B, CELF2, LZTS3, MYO3A, BPIFB4, BPI, SLC4A11, PHF20, TBC1D20, SIGLEC1, COMMD3, OPTN, MAP3K8, DYNLRB1, MYH7B, ANGPT4, TCF15, SPEF1, TCFL5, PARD3, BPIFB6, LBP, VIM, RNF24, SLC52A3, SPAG4, POFUT1, AAR2, PROCR, CDC123, NAA20, MCM10, DNAAF9, MAP1LC3A, DLGAP4, KIAA1217, CTNNBL1, CNBD2, BPIFB3, ARMC3, DHX35, IDI1, ACSS2, KAT14, PITRM1, PTF1A, CEP250, DIP2C, GFRA4, SPAG6, PAX1, ASXL1, EPB41L1, TP53INP2, GPR158, NNAT, TLDC2, RALGAPB, RALY, BPIFB2, KIF3B, SNORD87, KCNS2, CRH, PPP1R42, CA3, IL7, ST18, DECR1, CA2, PDE7A, GRHL2, RIPK2, MRPS28, COPS5, TRHR, TMEM68, SLC30A8, OSGIN2, BCL9L, chi-mir-326, TIMM8B, OR2D3, ABTB2, NLRP14, OR10A4, ART1, OR5J2, ARRB1, ZNF215, PIH1D2, GDPD5, C11orf65, PAMR1, RDX, POU2AF1, CCDC73, IL18, TREH, POGLUT3, EXPH5, PAX6, ARHGAP42, KLHL35, SLC37A4, APIP, DDX6, IFT46, PGAP2, SNORA66, ELF3, TIMM17A, ZBTB17, NFASC, CASP9, PHLDA3, SUCO, CNIH3, IPO9, TNNI1, CEP104, TFB2M, LZIC, CNTN2, MEGF6, WRAP73, SMYD3, CSRP1, TNNT2, RNPEP, C1orf174, LRRC47, FASLG, ATP1B1, PACC1, AGMAT, OGFOD2, PITPNB, CFAP251, B3GNT4, SPATA5, ERP29, RNF215, LIF, CASTOR1, ARL6IP4, ASCC2, BRI3BP, FICD, EIF4ENIF1, AACS, BCL7A, TRAFD1, TBC1D10A, FGF2,

		SF3A1, OSM, ISCU, MLXIP, TMEM131L, LRRC43, PISD, MN1, MTRFR, P2RX2, CRYBB1, IL15, TLR2, FBRSL1, BEAN1, SNORD68, HSD11B2, ZNF432, PLEKHF1, CMIP, NPAS1, RIPOR1, SPACA4, CDK10, MC1R, SLC9A5, LRRC36, ZNF546, ACSF3, DPEP1, FUT2, PLEKHG4, SPTBN4, CDH5, SPG7, PSMC4, POP4, PLCG2, ZNF276, LTBP4, CDH15, MAP3K10, CMTM3, SPIRE2, ZNF613, JPH3, ITFG1, TCF25, ZNF614, GAS8, PPP1R15A, CPNE7, SULT2B1, CKLF, SPHK2, PLD3, ARHGAP35, CMTM2, FGF21, DEF8, EGLN2, HYDIN, SHKBP1, SAE1, SNRPA, ITPKC, OGFOD3, DPH1, CYBC1, TP53I13, ELP5, RAB40B, OMG, CSNK1D, EVI2B, SGSM2, CRYBA1, GUCY2D, HIC1, CPD, CHD3, TAOK1, ACAP1, SRR, ALOX15B, WDR45B, SLC2A4, NSRP1, SLC39A11, RUNDC3A, CNTROB, ACADVL, ANKRD13B, GRN, OVCA2, TM4SF20, FAM124B, USP37, ZBTB40, CUL3, UBE2E3, STAT1, PADI3, RNF25, TMEM185B, ACVR2A, FARSB, PADI6, CFAP65, FEV, PKP4, C1QL2, PADI1, GRHL3, CRYBA2, STAT4, GALNT13, PRKAG3, COL4A4, HTR2B, SNORD72, ELOVL7, LPCAT1, DEPDC1B, CD180, SEMA5A, CTNND2, RPL37, CDH12, MOCS2, PLCXD3, PRKAA1, SLC12A7, FASTKD3, NSUN2, TTC33, MED10, SLC6A3, RETREG1, DROSHA, MYO10, C5orf49, FANCI, TICRR, C14orf28, MIR9, GOLGA5, PPP4R4, TOGARAM1, RIN3, BRMS1L, UNC79, MESP2, WDR93, MKRN3, ABHD2, GABRA5, GABRG3, CHSY1, U4, RRP9, DCAF1, SCAP, HYAL2, MIR128-2, DENND6A, SFMBT1, PRICKLE2, CFAP20DC, FEZF2, IQCF1, SHQ1, TADA3, LRIG1, MAGI1, PCBP4, CACNA2D2, GLT8D1, ARPP21, CCDC12, CISH, TMEM115, CMTM8, C3orf18, NPRL2, HYAL3, OXSR1, NEK4, MYD88, ARF4, CAMK1, PBRM1, SETD2, SUCLG2, RASSF1, DLEC1, CMTM7, ZMYND10, DNAH12, ABHD14B, KIF9, HYAL1, NT5DC2, CIDEC, IQCF5, PSMD6, CTNNB1, RPUSD3, RYBP, SLC22A13, NAA80, IFRD2, ZBTB22, APOM, UQCC2, SMIM29, RGL2, TAPBP, RXRB, LY6G6D, MPIG6B, RNF144B, HSP70.1, BAK1, HIVEP1, SPDEF, PXT1, LY6G5B, WDR46, SAPCD1, FAM83B, LY6G6F, ID4, IP6K3, VPS52, AIF1, NUDT3, KHDRBS2, PRRC2A, RPS10, NOL4, RAB31, VAPA, LDLRAD4, RNMT, ME2, chi-mir-187, WDR7, U1, NEDD4L, RTTN, KCTD1, ELAC1, CTIF, RAB27B, ESCO1, MC5R, PHLPP1, GNAL, IMPA2, CIDEA, ABHD3, DCC, SNORD14, CRAMP1, MIR25, AHSP, FUS, SNORA30, RNF40, TMEM204, chi-mir-106b, POLR2J2, chi-mir-93, JPT2,

		GIGYF1, SNN, VPS35L, ITGAL, PRR14, TGFB1I1, CALN1, DTX2, KCTD7, SLC5A2, ITGAM, CLEC16A, CTF1, TNFRSF17, UBFD1, CRCP, PLOD3, C7orf61, KDM8, TSC22D4, ACTL6B, ZNF646, SMURF1, PPP1R35, ZNF3, GPC2, KAT8, ZNF771, CIITA, ACHE, UFSP1, SEC14L5, ALKBH4, PHKG2, TPST1, MAPK8IP3, TRAPPC14, TRIM72, ITGAD, EPHB4, SNX29, FIS1, BCKDK, ERCC4, TMEM248, ZCWPW1, UBN1, PDAP1, NUBP2, FBXL19, VKORC1L1, TMEM130, GAL3ST4, KPNA7, SUMF2, ARMC5, UPK3B, CLDN15, MCM7, TAF6, SYCE1, VAX1, SHTN1, HSPA12A, POLL, CYP17A1B, SMNDC1, CUEDC2, SFXN4, ATE1, ARL3, PDZD8, ACTR1A, CYP17A1A, BBIP1, PSD, EIF3A, BNIP3, TCERG1L, FGFR2, FBXW4, PRDX3, SNORA19, LBX1, NKX6-3, BRF2, ANK1, MIR486-1, ADAM2, FGFR1, RARB, NR1D2, ASB5, RPL15, GPM6A, GOT1L1, CSMD1, ANGPT2, SPATA4, OXSM, ZMAT4, TM2D2, RAB11FIP1, PRF1, TMEM26, RHOBTB1, C1orf198, CCSAP, COMTD1, ERCC6, GDF10, TTC13, PGBD5, RBP3, FAM170B, DUSP29, CTNNA3, DRGX, GDF2, VSTM4, DUSP13, DNAJC12, SLC16A9, NUP133, RPS6KA4, PAK1, PRDX5, PHLDA2, CDKN1C, KCNJ5, CTSC, OPCML, ESRRA, FLI1, NUDT22, NAP1L4, GPR137, CCDC88B, PLCB3, BARX2, CLNS1A, DNAJC4, FERMT3, RAB38, OSBPL5, VEGFB, KCNK4, GJB3, ACKR1, GPR88, RAVER2, GJA4, PDE4B, HIPK1, TSHB, SLC5A9, MIGA1, CADM3, GJB4, GJB5, SETDB1, ZNF687, NFIA, SGIP1, VANGL1, PRUNE1, STXBP3, STRIP1, CACHD1, PTGFR, MAN1A2, SLC35A3, LURAP1, CDC14A, FNBP1L, TRMT13, DNAJB4, RCSD1, NEXN, MIER1, FAM102B, TSPAN1, FNDC7, UBE2U, ADGRL2, MAB21L3, PSMB4, SYCP1, NTNG1, RAD54L, RNPC3, SLC6A17, DMAP1, MFSD14A, SLC22A15, AHCYL1, LEPR, GIPC2, PATJ, USP40, DCLRE1B, ANXA9, AGL, NSUN4, AK5, GADD45A, DYNLT5, PSMD4, OLFML3, C8A, PALMD, HS2ST1, MSH4, POU2F1, LRRC7, CSF1, DNAJC6, OGDH, PON3, CDK6, KCP, TNPO3, PON1, RELN, OPN1SW, SUGCT, BRAF, HECW1, PALS2, DNAJC2, DENND2A, PON2, SLC26A5, NAPEPLD, AGAP2, OTOGL, MIR331, 7SK, INTS13, U5, STYK1, ZBED4, ERGIC2, PHETA2, TMTC3, PTHLH, MAPK8IP2, NCAPH2, RACGAP1, SMUG1, CHPT1, CYP27B1, SLC38A2, TM7SF3, RAPGEF3, MANSC1, ANO4, RPAP3, C12orf29, A2ML1, HDAC7, GLYCAM1, SPRYD3, PPP1R1A, PANX2, BCDIN3D, TCP11L2, CBY1, LRP6,

		KRT79, ITGB7, TMEM263, GTPBP1, DDX11, METTL1, WBP2NL, IQSEC3, ITPR2, AVIL, TRABD, OVCH1, SHANK3, CRACR2A, PIM3, NRIP2, MOV10L1, KRT8, FAIM2, RIC8B, AAAS, DENND6B, SYT10, DENND5B, CDK4, SMDT1, SELENOO, MIOX, TMTC1, RFX4, RIMKLB, VEZT, RARG, CYTH4, PHC1, ADM2, FOXM1, FBXO7, USP18, KRT78, CBX5, TOMM22, CSAD, KRT4, PTPRQ, B4GALNT1, MYBPC1, BTBD11, SP7, C4orf48, UNC5C, LAP3, AFP, GPRIN3, AFF1, NSD2, PITX2, TIFA, CWH43, CDS1, NCAPG, CABS1, SLC9B2, SLAIN2, USO1, TNIP3, TBC1D19, NSUN7, ODAM, TBC1D14, NAT8L, AMTN, NDNF, CCNA2, ENAM, CENPE, ZAR1, ALB, BDH2, PRDM8, SLC9B1, TACR3, AFM, ADGRL3, PTPN13, BBS7, HPGDS, NIPAL1, NPFFR2, DCUN1D4, FGF5, MAD2L1, LEF1, GPAT3, UBE2D3, SEC24D, SLC10A4, AMBN, MED28, QRFPR, MRPL4, MIR874, DBN1, F12, ICAM4, SNX2, SAFB, PFN3, TIFAB, CDKL3, DDX41, SIL1, C1QTNF2, GAMT, KLF16, NRG2, PFDN1, DOK3, PDLIM7, TCF3, MEX3D, CHAF1A, KLHL3, NEUROG1, RPL36, AFF4, C19orf25, ICAM1, FAM13B, HAVCR2, ABHD17A, KDM4B, PCSK4, FCHO1, PHAX, HDGFL2, CCNJL, CLK4, LMAN2, TEX43, MAP1S, LECT2, PDE4A, MACROH2A1, ZCCHC10, SPOCK1, HBEGF, HTR4, FSTL4, HSD11B1L, PPP2CA, FAM193B, MBD3, ICAM5, CDC25C, PRDM6, CSNK1G3, BTBD2, GRAMD2B, B3GNT3, ARRDC3, CDKN2AIPNL, DOCK5, PGM5, NFIL3, TRIM32, DCAF10, RFX3, MYT1L, PXDN, SPTLC1, PAPPA, NIPSNAP3A, AUH, CNTRL, INVS, PLGRKT, CDCA2, INSL6, VPS13A, ERCC6L2, OR13D1, KIAA2026, CTSB, ERMP1, ENPP3, SNORA33, SNORD101, CLDN20, SNORD100, COL10A1, GABRR1, FAXC, LAMA2, MAP3K7, TBX18, AIG1, ECT2L, TMEM200A, TSPYL1, GABRR2, ESR1, MTO1, MCHR2, SERINC1, SRSF12, RPS12, CCDC170, MEI4, TIAM2, OPRM1, RNGTT, CCDC28A, ATG5, ARID1B, AKAP7, MDN1, TRAPPC3L, EYA4, ARG1, GJE1, LYRM2, ADGRG6, L3MBTL3, ASCC3, SAMD5, GRM1
XP-$EHH_{JG\ to\ BG}$ > 0.121	880	3BHSD, 5S_rRNA, 7SK, AAMP, AASS, ABCA13, ABCD3, ABCD4, ABHD2, ABHD3, ABTB2, ACADL, ACAN, ACKR1, ACSS2, ADAM12, ADAM33, ADAMTSL4, ADARB2, ADCY3, ADGRF1, ADGRF5, ADGRG5, ADGRL2, ADM, ADRA1D, ADSS2, AK6, AKT3, ALCAM, ALKBH4, AMPD3, ANGEL2, ANKRD44, ANXA10, ANXA9, AP3B1, AP4E1, AP4S1, APC, AQP4, AREL1, ARG1, ARHGAP10, ARHGAP15, ARHGAP25, ARHGDIG,

		ARL6, ARL9, ARMC6, ARMH4, ARRB1, ART1, ASPH, ATE1, ATF5, ATG4C, ATP1B1, ATP5F1C, ATP5PB, ATP6V0D2, AVPR1A, AZIN2, B3GALNT2, B3GNT2, B3GNT3, BARX2, BATF3, BBX, BCKDHB, BDH2, BEST3, BET1, BIRC6, BMP2, BNC2, BNIP3, BNIP5, BPIFB2, BPIFB3, BPIFB4, BPIFB6, BRMS1L, BTBD19, BTBD8, C10orf82, C11orf42, C11orf97, C1orf112, C1orf115, C1orf146, C1orf162, C1orf198, C1orf68, C1QL2, C1R, C1RL, C20orf27, C2orf88, C4orf48, C5AR1, C9, C9orf72, CA13, CAAP1, CACHD1, CADM2, CADM3, CADPS2, CAMTA2, CAPN15, CAPZA2, CASP3, CASP6, CASR, CCDC125, CCDC146, CCDC175, CCDC185, CCDC192, CCDC24, CCDC28A, CCDC61, CCDC71L, CCDC9, CCL14, CCL17, CCL22, CCNA1, CCR8, CCSAP, CD109, CD180, CD86, CEACAM16, CEACAM19, CEBPB, CENPB, CENPU, CEP126, CEP128, CEP135, CEP170, CER1, CERT1, CETN1, CFAP300, CFAP54, CFAP95, CFTR, CHEK1, chi-mir-107, chi-mir-187, chi-mir-326, chi-mir-582, chi-mir-876, CHMP5, CHRNA5, CHRNA6, CHST9, CLDN15, CLDN20, CLPB, CLUL1, CMC1, CMSS1, CNGA4, CNIH3, COCH, COL10A1, COMMD1, COQ8A, COQ9, CPA6, CPNE3, CPNE5, CPNE8, CPQ, CPXM1, CRABP1, CRAMP1, CREB3L2, CREB5, CRH, CROT, CRPPA, CRTAC1, CRX, CRYGN, CSF1R, CSRP1, CST7, CTH, CTNNA3, CTNNBL1, CTSC, CTXN1, CYP39A1, CYP4B1, CYP7B1, DAB2, DCAF1, DCP2, DDHD1, DDX20, DECR2, DHRS7, DMAP1, DMBX1, DMXL1, DNAH14, DNAJA4, DNAJB14, DNAJC2, DNAJC25, DNASE1L3, DNER, DNHD1, DNM3, DNPEP, DOCK9, DPY19L4, DSEL, DTWD1, DTX2, DUSP11, DYDC1, E2F5, EAF2, ECT2L, EDIL3, EDN1, EDNRB, ELAVL1, ELAVL4, ELF5, ELK3, ELMOD2, ELOVL6, ELSPBP1, EMILIN2, EN1, ENPP3, ENPP5, ENPP6, ENTPD6, EPHX4, EPS8, EQTN, ERCC4, ESCO1, ESRP1, ETFDH, EXO5, EXOC3L4, EXOC5, EXOSC8, EYA1, EYA2, FAHD1, FAM120A, FAM135A, FAM168B, FAM177A1, FAM234A, FAM83B, FAM83F, FANCF, FANCI, FAT1, FBH1, FBN3, FBXL13, FBXL17, FBXO22, FBXW4, FCHO1, FERD3L, FGF14, FGF21, FGF5, FGF7, FGF8, FIS1, FN1, FREM1, FRK, FUT2, FUT4, FYB1, FYCO1, G2E3, GAB2, GADD45A, GAL3ST4, GCNT4, GDAP1L1, GDF10, GDF2, GDPD5, GFRA4, GHSR, GIPC2, GJC3, GLRB, GMFB, GMPR2, GNG4, GOLGA4, GORAB, GPAM, GPC2, GPD1L, GPR135, GPR18, GPR183, GRAP2, GRIA2, GSX2, GTF3C3, GYS2, HAO2, HBAI, HBM, HBQ1, HDLBP,

		HEATR1, HECW2, HIF3A, HIVEP1, HLX, HMCN1, HNF4A, HPDL, HSDL2, HSPA12A, HYKK, IDH3A, IDI1, IFI44, IFNE, IL12RB2, IL15, IL18, IL19, IL20, IL23R, IL24, IL6ST, ILK, ILRUN, INAFM1, INCA1, INO80C, INSM2, INTS8, IPO7, IREB2, IRF2, IRX2, ITGA9, ITGB1, ITIH2, IYD, JARID2, JPT2, JRKL, KCNE3, KCNH5, KCNIP2, KCNK10, KCNQ5, KCTD1, KDM4A, KHDRBS2, KIF18A, KIF2C, KIF6, KLF12, KLF17, KLF2, KLHL35, KMT2C, KPNA7, KPRP, KRT4, KRT78, KRT79, KRT8, L3HYPDH, LBHD2, LCE3B, LDLRAD4, LEPR, LIMCH1, LIN28A, LIPI, LIPT2, LMAN2, LPCAT3, LRP6, LRRC1, LRRC19, LRRC7, LRRCC1, LRRTM1, LTA, LTB4R, LTB4R2, LTBP2, LYST, LZIC, MACF1, MAGI3, MAN1A2, MANBA, MANBAL, MAP1S, MAPK8IP3, MARK1, MAT1A, MB21D2, MBOAT2, MC5R, MCCD1, MCHR2, MCRIP2, MDFIC, MDH2, ME3, MEGF11, MESP2, MET, Metazoa_SRP, MFSD14B, MGAT1, MIR153, MIR31, MIR3120, MIR760, MIR9, MLLT3, MOB3B, MOCS2, MPG, MSH4, MSL2, MTARC2, MTCH1, MTFMT, MTHFD1L, MTNR1A, MUC15, MXD3, MYH14, MYL1, MYL10, NALCN, NAMPT, NAPEPLD, NARS2, NAT8L, NCAN, NCOA2, NDC80, NDE1, NDUFB10, NECTIN1, NEDD1, NFATC4, NFE2L3, NFKBIL1, NFX1, NHLRC4, NINL, NLRP14, NME4, NNAT, NOP9, NOX4, NPAS1, NPAS3, NPC2, NPY, NR5A2, NRCAM, NSD2, NSL1, NTMT2, NUBP2, NUBPL, NUDT12, NUDT9, NUF2, NUP133, ODR4, OGA, OR10D3, OR11H4, OR11H6, OR11H7, OR13G1, OR2AE1, OR2Y1, OR51I2, OR52E5, OR52N2, OR52N4, OR52W1, OR56B4, OTOGL, OTUD3, OTUD7A, OXTR, P4HA3, PACC1, PAG1, PANK1, PAPPA, PARD6G, PARVA, PASK, PCDH7, PCED1A, PDAP1, PDE2A, PDE4B, PDE6A, PDE7A, PDE7B, PDE9A, PDGFRA, PDGFRB, PDIA2, PDP1, PGAP1, PGAP2, PGBD1, PGBD5, PGM2L1, PHB2, PHF2, PHLDA3, PHOX2A, PICALM, PIGQ, PIH1D2, PIP5K1B, PITRM1, PITX2, PLA2G12A, PLA2G4A, PLA2G5, PLA2G7, PLAA, PLAT, PLBD1, PLD5, PLEKHA8, PLEKHG1, PLK3, PLOD3, PMP2, POC5, PODN, POLL, POLR2B, POLR2J2, POLR3A, PPIL1, PPP1R14C, PPP1R15A, PPP3CA, PPP4R4, PPP5C, PRDM14, PRDM8, PRF1, PRG4, PRKAG2, PRKAG3, PROKR1, PROX2, PRPH2, PRR15, PRR16, PRR35, PRRX1, PRSS35, PRUNE1, PTCHD4, PTDSS1, PTGER2, PTHLH, PTPN21, PTPRF, PTPRN, PTPRQ, PTRHD1, PUM2, PURG, PUS7L, PVR, PXT1, R3HCC1, R3HDML, RAB11FIP3, RAB24, RAB38,

		RAB40C, RAD18, RAG2, RAP1A, RAPH1, RASL12, RAVER2, RBMS3, RBP3, RBP5, RCAN2, REC8, RECQL, RELN, REV3L, RFXANK, RGS4, RHBDL1, RHEB, RHOT2, RIMS1, RLF, RNF114, RNF151, RNF170, RNF24, RNF25, RNMT, ROR1, ROS1, RPAP2, RPAP3, RPGRIP1, RPS8, RRM1, RRP15, RXFP1, RYK, SAE1, SCARNA12, SCARNA18, SCFD1, SCML4, SCN2A, SCOC, SCP2, SCTR, SEC14L5, SEC31B, SEC63, SEL1L, SEMA5A, SERPINB11, SERPINB13, SERPINB7, SERTM1, SETDB1, SF3B1, SI, SIGLEC1, SIMC1, SIX3, SLC15A1, SLC16A12, SLC16A9, SLC20A2, SLC24A2, SLC25A38, SLC25A42, SLC26A5, SLC35D3, SLC37A1, SLC38A2, SLC38A4, SLC38A9, SLC39A11, SLC4A5, SLC5A12, SLC9B1, SLC9B2, SLCO1A2, SLCO4C1, SLCO5A1, SLCO6A1, SLITRK1, SLITRK3, SMAP2, SMCHD1, SMG7, SMURF1, SNAI1, SNAP47, SNORA23, SNORA25, SNORA3A, SNORA3B, SNORA66, SNORA70, SNORA72, SNORA8, SNORD38A, SNORD38B, SNORD39, SNORD45A, SNORD5, SNORD6, SNRNP25, SNTB1, SNX3, SOLD1, SOSTDC1, SOX5, SPACA4, SPAG7, SPATA45, SPDL1, SPEF1, SPG21, SPHK2, SPIDR, SPINK4, SRC, SRGAP2, SRP19, SRP54, SRP72, SRSF11, SS18, ST3GAL3, STIM1, STK24, STPG2, STT3A, STT3B, STUB1, SULT2B1, SUPT3H, SWT1, SYCE1, SYCP2L, SYNDIG1, SYNDIG1L, SYPL1, SYT11, SYT9, TAB2, TACR3, TAF1D, TAF3, TBC1D14, TBC1D23, TBCC, TBCK, TBL3, TCERG1L, TCF19, TDRD7, TEAD1, TEAD3, TELO2, TESPA1, TEX38, TG, TGFA, THAP1, THEGL, TIAM2, TICRR, TIGD6, TIMM8B, TKTL2, TLL1, TLR2, TLX3, TMCO2, TMEM130, TMEM131L, TMEM182, TMEM196, TMEM204, TMEM209, TMEM229A, TMEM30B, TMOD1, TMTC3, TNF, TNFRSF21, TNFRSF9, TNFSF10, TNNI1, TNNT2, TOE1, TOM1L1, TOX4, TP53INP2, TPRKB, TRAF6, TRAPPC14, TRIM4, TRMT5, TRPA1, TSPYL1, TSSK4, TTC13, TTC21A, TTC27, TTK, TTPAL, TUB, TULP1, TWF1, TYMS, U1, U2, U5, U6, U7, UBE2D3, UBE2E2, UBE2E3, UBE2Q2, UBN1, UCHL1, UCK2, UGP2, UPK3B, UROD, USP37, USP38, USP40, UTS2, VOPP1, VPS35L, VSTM5, VWA5A, VWC2, WDR48, WDR86, WDR90, WDR93, WFIKKN1, WIPF3, WWP1, WWTR1, XCR1, XYLT1, Y_RNA, YIPF4, ZAP70, ZCWPW1, ZDHHC21, ZFP62, ZFP69, ZFYVE1, ZIC5, ZKSCAN4, ZKSCAN8, ZMPSTE24, ZMYM4, ZNF143, ZNF165, ZNF232, ZNF24, ZNF394, ZNF473, ZNF684, ZNF704, ZNF710, ZSCAN16, ZSCAN26, ZSWIM9
