# Supplementary material for: Multiomics analyses of Jining Grey goat and Boer goat reveal genomic regions associated with fatty acid and amino acid metabolism and muscle development
Source: Anim Biosci. 2023 Nov 2;37(6):982–92. doi: 10.5713/ab.23.0316 (PMC11065957; doi:10.5713/ab.23.0316)
Supplement: Supplementary file 7 [file ab-23-0316-Supplementary-Fig-1,2.pdf]

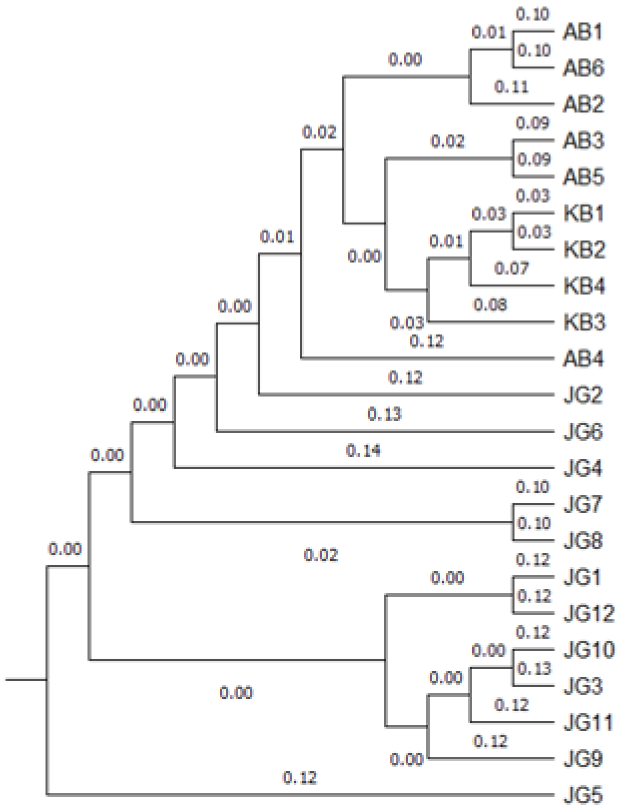

Supplementary Figure 1. Phylogenetic tree of 22 individuals. AB is short for Australian Boer Goat. KB is short for Korean Boer Goat. JG is short for Jining Grey Goat.

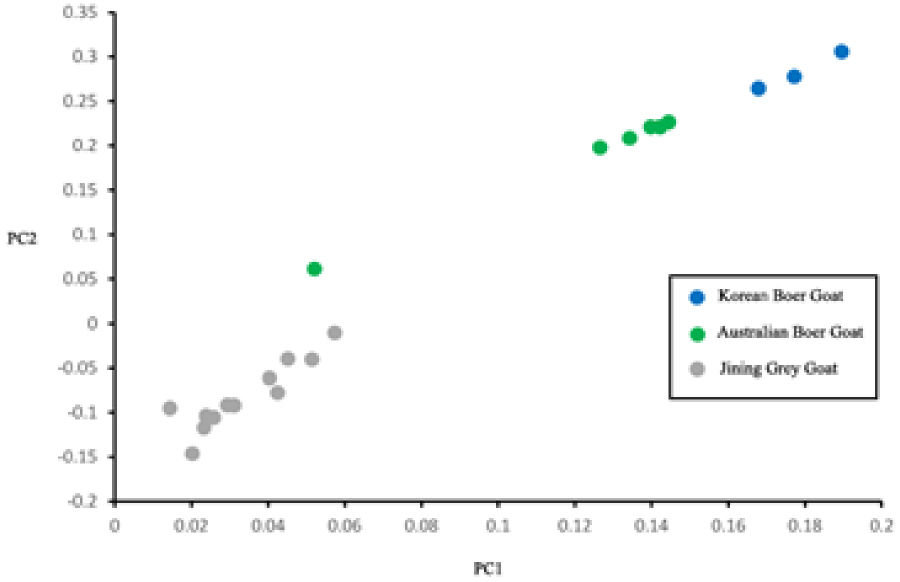

Supplementary Figure 2. Principal component analysis of 22 individuals.
